# Telomere transcripts act as tumor suppressor and are associated with favorable prognosis in colorectal cancer with low proliferating cell nuclear antigen expression

**DOI:** 10.1007/s13402-024-00986-y

**Published:** 2024-09-02

**Authors:** Philip Kienzl, Abigail J. Deloria, Monika Hunjadi, Juliane M. Hadolt, Max-Felix Haering, Angrit Bothien, Doris Mejri, Medina Korkut-Demirbaş, Sandra Sampl, Gerhard Weber, Christine Pirker, Severin Laengle, Tamara Braunschmid, Eleni Dragona, Brigitte Marian, Sarantis Gagos, Lingeng Lu, Jeremy D. Henson, Loretta M. S. Lau, Roger R. Reddel, Wolfgang Mikulits, Stefan Stättner, Klaus Holzmann

**Affiliations:** 1 Center for Cancer Research, Comprehensive Cancer Center, Medical University of Vienna, Borschkegasse 8a, Vienna, A-1090 Austria; 2 Present Address: Department of Dermatology, Medical University of Vienna, Vienna, Austria; 3 Present Address: Center for Medical Physics and Biomedical Engineering, Medical University of Vienna, Vienna, Austria; 4 Department of Cardiac Surgery, Medical University of Vienna, Vienna, Austria; 5 Department of Surgery, Social Medical Center South, Kaiser Franz Josef Hospital, Vienna, Austria; 6 Present Address: Department of Surgery, Klinik Floridsdorf, Wiener Gesundheitsverbund, Vienna, Austria; 7 Laboratory of Genetics Center of Clinical Research, Experimental Surgery and Translational Research, Biomedical Research Foundation of the Academy of Athens, Greece (BRFAA), Soranou Efesiou 4, Athens, 115 27 Greece; 8 Department of Chronic Disease Epidemiology, Yale School of Public Health, School of Medicine, Yale Cancer Center, Yale University, New Haven, USA; 9 Prince of Wales Clinical School, University of NSW, UNSW, Sydney, 2052 Australia; 10 Children’s Cancer Research Unit, The Children’s Hospital at Westmead, Faculty of Medicine and Health, University of Sydney, Westmead, 2145 Australia; 11 Children’s Medical Research Institute, Faculty of Medicine and Health, The University of Sydney, Westmead, 2145 Australia; 12 Present Address: Department of General, Visceral and Vascular Surgery, Salzkammergut Klinikum, OÖG, Dr. Wilhelm Bock Strasse 1, Vöcklabruck, 4840 Austria

**Keywords:** Telomere and proliferation parameters, Tumor progression, Colorectal cancer survival, Correlation study

## Abstract

Telomeric repeat-containing RNAs (TERRA) and telomerase RNA component (TERC) regulate telomerase activity (TA) and thereby contribute to telomere homeostasis by influencing telomere length (TL) and the cell immortality hallmark of cancer cells. Additionally, the non-canonical functions of telomerase reverse transcriptase (TERT) and TERRA appear to be involved in the epithelial-mesenchymal transition (EMT), which is important for cancer progression. However, the relationship between TERRA and patient prognosis has not been fully characterized. In this small-scale study, 68 patients with colorectal cancer (CRC) were evaluated for correlations between telomere biology, proliferation, and EMT gene transcripts and disease outcome. The proliferating cell nuclear antigen (PCNA) and the epithelial splicing regulatory proteins 1 and 2 (ESRP1 and ESRP2) showed a positive correlation with TERRA, while TA and TERRA exhibited an inverse correlation. Consistent with previous findings, the present study revealed higher expression levels of TERT and TERC, and increased TA and TL in CRC tumor tissue compared to adjacent non-tumor tissue. In contrast, lower expression levels of TERRA were observed in tumor tissue. Patients with high TERRA expression and low PCNA levels exhibited favorable overall survival rates compared to individuals with the inverse pattern. Furthermore, TERRA suppressed CRC tumor growth in severe combined immunodeficiency disease (SCID) mice. In conclusion, our study extends previously published research on TERRA suggesting its potential therapeutic role in telomerase-positive CRC.

**Supplementary Information:**

The online version contains supplementary material available at 10.1007/s13402-024-00986-y.

CRC ranks as the third most common cancer globally with high mortality rates [1]. Even though a considerable improvement of therapy options could be seen during the last decades, prediction of patient’s individual prognosis is still challenging [2]. So far, the study of coding genes has successfully advanced our knowledge of cancer biology [3]. More recently, regulatory roles of several long noncoding RNA (lncRNA) transcripts have been identified as significantly implicated in cancer hallmarks, including the facilitation of replicative cancer cell immortality [4]. LncRNAs such as TERC and TERRA regulate immortality by maintaining TL and preventing senescence in cancer cells [5]. TERRA plays critical roles in telomere homeostasis by regulating TA at telomeres and influencing TL both in vitro and in vivo [6–9]. In addition, the expression of TERRA was associated with the proliferative state of the cell [10, 11]. So far, one translational study investigated TERRA expression in CRC and showed that high TERRA levels and low carcinoembryonic antigen levels before surgery were associated with improved patient disease free survival [12]. There is accumulating evidence that the telomerase enzymatic subunit TERT possesses further functions independent of telomere maintenance in the tumor and the microenvironment, including induction of EMT which is a hallmark of tumor progression [13–17]. For example in CRC, TERT with the zinc finger E-box binding homeobox 1 (ZEB1) transcription factor forms a complex that binds to the promoter of E-cadherin to repress its expression and induce EMT [18]. Furthermore, snail family transcriptional repressor 1 (Snail1) another important EMT regulator negatively regulates TERRA and represses TERT in mice [19]. This indicates that the interactions between TERT, TERRA and tumor aggressiveness are complex and the in vivo evidence of correlations between telomere and EMT parameters and the relation to CRC outcome are not well established.

This translational study employed matched pairs of tumor (T) and adjacent non-tumor (N) tissue from CRC patients to determine individual level differences as T/N ratios in telomere (TL, TA, TERT, TERC, TERRA) and proliferation (PCNA) parameters and extends previously published research on ESRP1 and ESRP2 [20].

TL, TA, and the mRNA expression profiles of TERT, TERC and PCNA, as well as the TERRA lncRNA expression profiles were analyzed in the tissue of 68 CRC patients (Fig. 1 and Supplementary Table 1). The median value of TL in kbp was slightly higher (1.18-fold,) in T than N tissue (Fig. 1A). Furthermore, TA in total product generated (TPG) units and relative quantities (RQ) of TERT, and TERC exhibited significant increases in T compared to N tissue, with median fold changes of 11.5-, 1.1-, and 2.1-, respectively. Mean TA in N tissue was 2.12 TPG units, less TA was identified in 7 of 68 (10%) CRC cases in the T tissue that may not use TA as telomere maintenance mechanism. Notable, these 7 CRC cases compared to remaining 61 cases showed a 5.1-fold higher median level for pan-chromosomal (p.c.) TERRA expression in T but not in N tissue with p-values 0.0061 and 0.323, respectively (Supplementary Fig. 1). Furthermore, a 14.7-fold reduction in the median of p.c.TERRA expression was evident in T compared to the N tissue of all CRC cases studied (Fig. 1A). Consistent with the alteration in the p.c.TERRA level, TERRA originating from chromosomes 2p and 18p showed median reductions of 10.7- and 15.0-fold in T compared to N tissue, respectively. Compared to others, 2p and 18p are most similar to p.c.TERRA expression in T tissue and as a T/N ratio identified by screening of 5 CRC cases (Supplementary Fig. 2). PCNA was selected as marker for proliferation from a panel of five proliferation-associated genes Ki-67 (MKI67), MYC proto-oncogene (MYC), cyclin D1 (CCND1), cyclin A2 (CCNA2), and PCNA evaluated by a pilot experiment for correlation with p.c.TERRA in T tissue and T/N ratios (Supplementary Fig. 3). Median values of PCNA expression remained indistinguishable between T and N tissue (Fig. 1A).

**Fig. 1:**
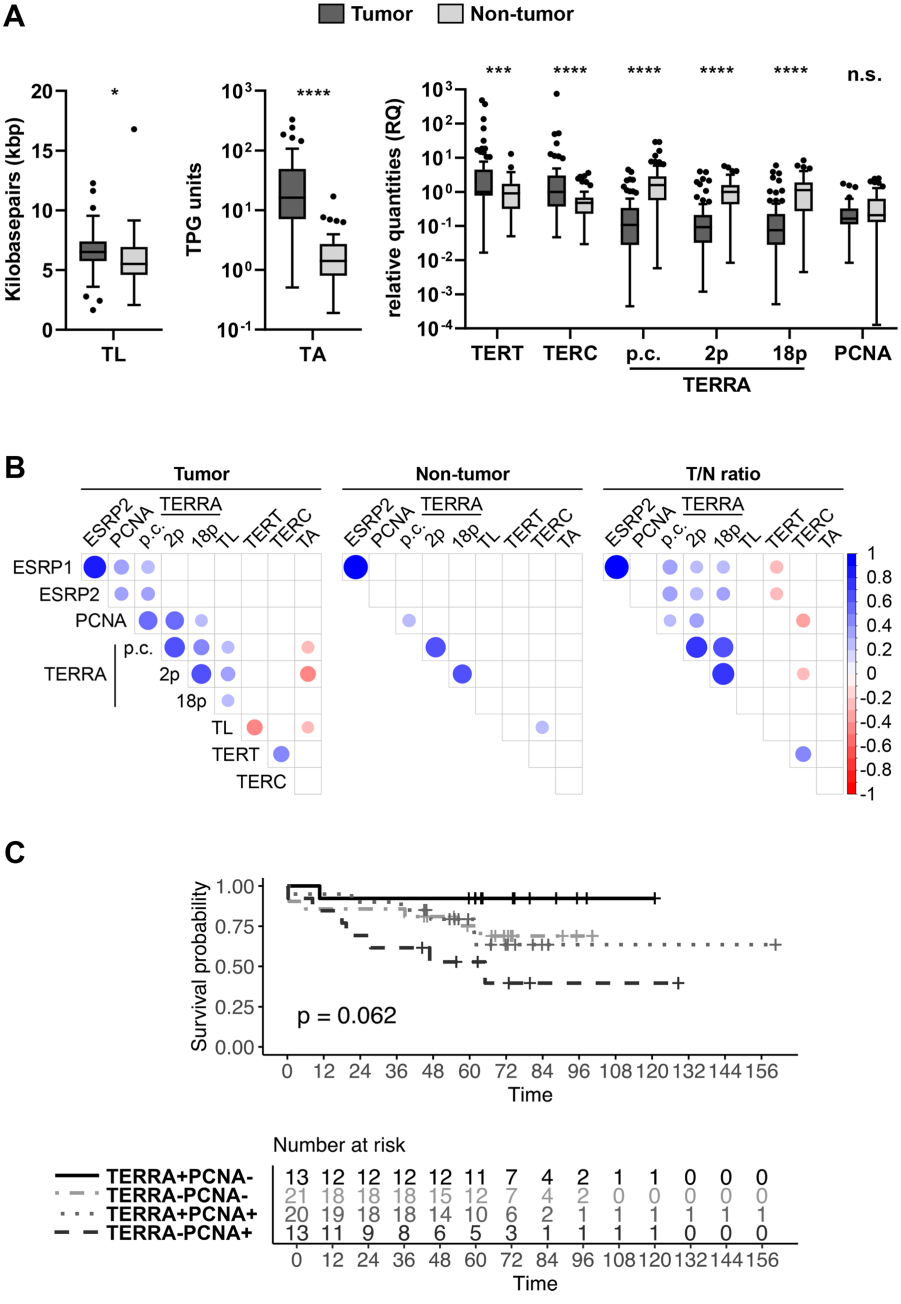
CRC patient study. **A** Screening of tumor (T) and adjacent non-tumor (N) tissue from CRC cases (*n* = 68). Boxes show median and interquartile range with individual values overlaid. Telomere length (TL) in kbp, telomerase activity (TA) in total product generated (TPG) units, and relative quantity (RQ) values for telomerase reverse transcriptase (TERT), telomerase RNA component (TERC), pan-chromosomal TERRA (p.c.TERRA), chromosome-specific 2p TERRA (2p TERRA) and 18p TERRA (18p TERRA), and proliferating cell nuclear antigen (PCNA). For comparison of T and N samples, RQ values were log transformed and paired *t* test was applied. *p* < 0.05 (*), *p* < 0.001 (***), *p* < 0.0001 (****), *p* > 0.05 not significant (n.s.). **B** Correlation plots depicting relationships between the continuous variables including epithelial splicing regulatory protein 1, 2 (ESRP1, ESRP2) in tumor, non-tumor and T/N ratios. Pearson’s r is color-coded, and dots denote significant correlations, with strength represented by dot size (big, *p* < 0.0001; small, *p* < 0.05). **C** Overall survival (OS) rates of CRC patients by Kaplan-Meier analyses. Patients were grouped by median values of T/N ratios into four categories of cases with high and low p.c.TERRA and PCNA levels. The survival curves for each category are presented and differ with borderline significance (Log-rank *p* = 0.062). However, comparing survival rates of the two categories: low TERRA T/N and high PCNA T/N levels (TERRA−/PCNA+) and high TERRA T/N and low PCNA T/N levels (TERRA+/PCNA−) was significant (Log-rank *p* = 0.013). The x-axis represents OS time in months

Correlations between all parameters were analyzed and evaluated for significance (Fig. 1B). Correlations between telomere transcripts and proliferation were found. Notably, within T tissue, several positive correlations were observed, such as among p.c., 2p, and 18p TERRA and PCNA transcript levels with Pearson coefficients (*r*) of 0.58, 0.56 and 0.26. In adjacent N tissue, a positive correlation was observed for p.c.TERRA and PCNA (*r* = 0.29). The calculated T/N ratios revealed, that PCNA positively correlated with p.c. and 2p TERRA (*r* = 0.25, 0.34). Several connections between master splice regulators for EMT (ESRP1 and ESRP2) and other parameters were evident. T tissue and T/N ratios demonstrated positive correlations between p.c.TERRA and ESRP1, and TERRA and ESRP2 (T tissue: *r* = 0.30, 0.31; T/N ratios: *r* = 0.36, 0.37). When we considered T/N ratios also chromosome-specific 2p and 18p TERRA correlated with ESRP1 (*r* = 0.27, 0.28) and ESRP2 (*r* = 0.29, 0.30). PCNA correlated with ESRP1 and ESRP2 in T tissue (*r* = 0.37, 0.31). T/N ratios showed a weak, but significant negative correlation between TERT and ESRP1/ESRP2 (*r* = −0.29, −0.27), and a moderately negative correlation (*r* = −0.32) between TERC and PCNA.

In T tissue and T/N ratios, p.c.TERRA correlated with 2p and 18p TERRA (T tissue: *r* = 0.69, 0.42; T/N ratios: *r* = 0.75, 0.68). In N tissue p.c.TERRA and 2p TERRA showed a positive correlation (*r* = 0.67). Chromosome-specific 2p and 18p TERRA exhibited strong correlations when T, N tissue and T/N ratios were considered (*r* = 0.64, 0.60, 0.79). Both, p.c.TERRA and 2p TERRA, inversely correlated with TA in T tissue (*r* = −0.26, −0.41). We noticed several intriguing associations between telomere associated parameters A positive correlation between p.c., 2p and 18p TERRA and TL was found in T tissue (*r* = 0.27, 0.35, 0.26) whereas 2p TERRA and TERC correlated negatively in T/N ratios (*r* = −0.26). Surprisingly, no correlation between TA and telomerase subunits TERT or TERC was evident. Furthermore, another negative correlation was noted between TL and TERT expression in T tissue (*r* = −0.42) and between TL and TA (*r* = −0.25). In contrast, a positive correlation between TL and TERC was evident in N tissue (*r* =0.28). Finally, both T tissue and T/N ratios demonstrated a positive correlation between TERT and TERC (*r* = 0.43, 0.40) (Fig. 1B).

To gain insight into the relationship between studied parameters and patient outcome, we performed Cox regression analysis (Table 1). In univariate Cox models, PCNA expression in N tissue and T/N ratios were identified to be associated with patient overall survival (OS) and hazard ratios (HR) of 0.67 and 1.41, respectively. The multivariate Cox model was adjusted for age, stage, grade, tumor site, gender, microsatellite stability and confirmed the findings of the univariate model. In addition to the univariate model results, chromosome-specific 18p TERRA in N tissue and TA in T tissue and T/N ratios were identified to have independent effects on OS. In fact, the HRs of PCNA in N tissue and T/N ratios, chromosome-specific 18p TERRA in N tissue, TA in T and T/N ratios were 0.60, 1.50, 1.41, 0.70, 0.75, respectively.

**Table 1:** Cox proportional hazard model and *P* values of the likelihood ratio test

	*n*	Univariate			Multivariate*		
		HR	95% CI	*P* value	HR	95% CI	*P* value
**Tumor**							
ESRP1	68	0.82	0.49 – 1.37	0.457	0.82	0.45 – 1.51	0.159
ESRP2	68	0.78	0.44 – 1.37	0.393	0.85	0.44 – 1.67	0.168
PCNA	68	1.07	0.66 – 1.73	0.796	1.12	0.63 – 1.99	0.171
TERRA pan	68	0.91	0.75 – 1.10	0.331	0.81	0.63 – 1.03	**0.070**
TERRA 2p	68	1.12	0.88 – 1.43	0.371	1.15	0.85 – 1.54	0.140
TERRA 18p	68	1.01	0.82 – 1.25	0.934	1.03	0.80 – 1.33	0.176
TERT	67	0.88	0.70 – 1.10	0.236	0.87	0.68 – 1.10	0.118
TERC	67	0.89	0.69 – 1.14	0.339	0.88	0.68 – 1.15	0.143
TL	68	1.65	0.37 – 7.38	0.504	1.37	0.22 – 8.45	0.173
TA	68	0.76	0.57 – 1.01	0.062	0.70	0.52 – 0.95	**0.034**
**Non-tumor**							
ESRP1	67	1.08	0.80 – 1.46	0.600	0.95	0.69 – 1.31	0.143
ESRP2	67	1.10	0.81 – 1.49	0.525	0.99	0.72 – 1.38	0.147
PCNA	67	0.67	0.50 – 0.89	**0.015**	0.60	0.42 – 0.84	**0.014**
TERRA pan	67	1.13	0.87 – 1.46	0.341	1.08	0.81 – 1.44	0.136
TERRA 2p	67	1.14	0.79 – 1.64	0.469	1.24	0.83 – 1.84	0.101
TERRA 18p	67	1.23	0.91 – 1.66	0.151	1.41	0.99 – 2.01	**0.035**
TERT	67	1.12	0.76 – 1.63	0.567	1.12	0.75 – 1.67	0.134
TERC	67	1.02	0.68 – 1.55	0.919	1.04	0.70 – 1.55	0.145
TL	67	0.96	0.28 – 3.38	0.954	0.75	0.18 – 3.05	0.140
TA	67	0.84	0.53 – 1.34	0.474	0.86	0.54 – 1.37	0.129
**T/N ratio**							
ESRP1	67	0.88	0.65 – 1.19	0.385	1.01	0.72 – 1.40	0.147
ESRP2	67	0.87	0.64 – 1.18	0.337	0.99	0.72 – 1.35	0.147
PCNA	67	1.41	1.09 – 1.82	**0.019**	1.50	1.12 – 2.02	**0.019**
TERRA pan	67	0.81	0.64 – 1.02	**0.062**	0.82	0.65 – 1.04	**0.053**
TERRA 2p	67	1.04	0.84 – 1.28	0.738	1.01	0.80 – 1.28	0.147
TERRA 18p	67	0.86	0.67 – 1.12	0.242	0.86	0.67 – 1.11	**0.091**
TERT	66	0.85	0.69 – 1.04	0.117	0.83	0.65 – 1.04	**0.063**
TERC	66	0.88	0.68 – 1.14	0.307	0.87	0.66 – 1.15	0.111
TL	67	1.37	0.49 – 3.82	0.550	1.62	0.45 – 5.84	0.123
TA	67	0.78	0.58 – 1.06	0.116	0.75	0.56 – 0.99	**0.042**
**Metastasis**	67	2.38	0.79 – 7.18	0.158			

*adjusted for age, stage, grade, tumor site, gender, microsatellite stability

*HR* hazard ratio, *CI* confidence interval, *P* values < 0.1 are marked in bold

Next, the combined impact of TA and TERRA/PCNA transcript levels on OS in our patient cohort was evaluated (Fig. 1C). The stratification of patients into four groups based on their high and low values above or below the median cutoff for TA and PCNA, as well as for TA and p.c.TERRA, did not result in any significant influence on OS (log-rank *p*-values = 0.24) (Supplementary Fig. 4). In contrast, stratification based on TERRA and PCNA values resulted borderline significance (Log-rank *p* = 0.062). Furthermore, two of four groups with the categories, high p.c.TERRA and low PCNA were associated with a favorable patient outcome compared to low p.c.TERRA and high PCNA (Fig. 1C). Notably, the demographic characteristics between these two groups were similar for age, tumor stage, grade, tumor site, microsatellite stability and gender (Supplementary Table 2). Taken together, these data suggest that levels between tumor and adjacent non-tumor tissue of TERRA and PCNA can influence CRC patient survival.

A panel of eight CRC cell lines was used to analyze telomere parameters (Fig. 2A). Cell lines were screened for TL and TA levels in TPG units. All CRC cell lines demonstrated TA but one subline of SW480 termed SW480-LT showed the highest telomeric content and longest telomeres (>20 kbp) as validated by TRF analyses (Supplementary Fig. 5). Furthermore, SW480-LT cells demonstrated high p.c.TERRA transcript expression levels (Fig. 2A). In addition, among the studied CRC cell lines, SW480-LT exhibited the highest expression of chromosome-specific TERRA. The isogenic long telomere (LT) model was derived in vitro as a spontaneous outgrowth from SW480 and demonstrated increased growth rate with altered cell characteristics such as morphology and size (Supplementary Fig. 6). In addition, SW480-LT cells showed extremely low ESRP1 expression and were previously characterized to demonstrate a shift from an epithelial to a more mesenchymal phenotype with associated changes in EMT gene expression profiles [20]. LT cells showed increased intensity and number of telo-FISH signals in interphase nuclei (Fig. 2B). Compared to the parental SW480 cells that exerted major clones with karyotypes composed of 50–55 chromosomes and displayed whole genome duplication in 3–4% of co-dividing cells [21], the great majority of SW480-LT metaphases were composed from 100–110 chromosomes (Fig. 2C). In addition, the SW480-LT occasionally displays double minute chromosomes, hyper-polyploid cells of about 200–300 chromosomes and chromosome fusions in small percentages of metaphases (less than 2%). Inhibition of TA by ectopic TERT dominant-negative (dn) expression demonstrated increased numbers of senescent cells and that both SW480-LT and SW480 depend on TA as telomere maintenance mechanism to avoid replicative senescence (Fig. 2B). SW480-LT exhibited increased colony formation growth in 2D, but not 3D, similar to SW620 cells that were established from a metastasis of the same CRC patient (Fig. 2D). In addition, SW480-LT showed increased migration and invasion compared to SW480 and SW620 cells.

**Fig. 2:**
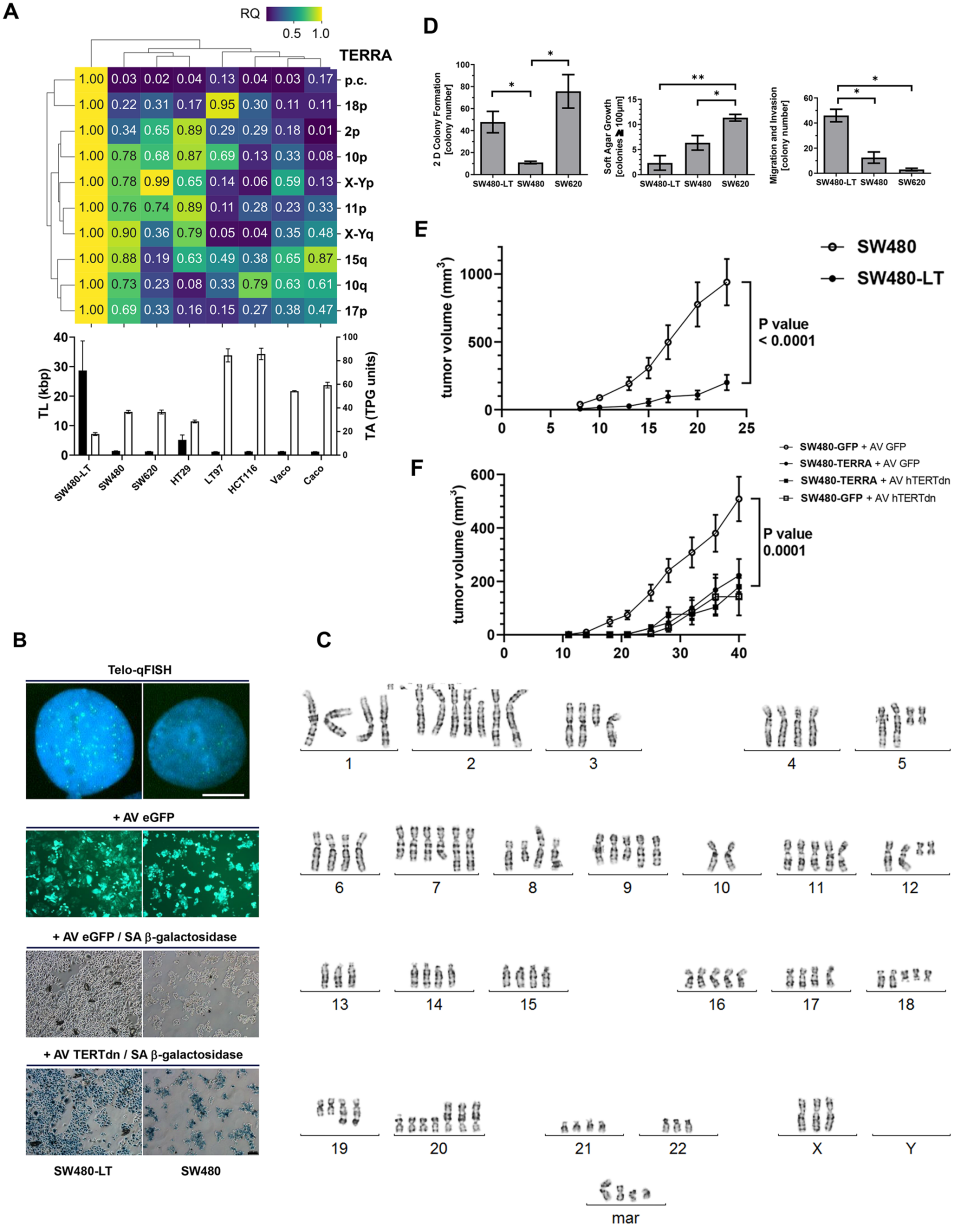
Effects of TERRA expression on CRC cell growth in vitro and as SCID mouse xenotransplants in vivo. **A** Screening of CRC cell lines (*n* = 8) for TERRA expression measured in RQ values, TA in TPG units and TL in kilobasepairs (kbp). SW480, its subline with long telomeres (LT) SW480-LT, SW620, LT97, HT29, HCT116, Vaco, and Caco-2 were studied. Bar plots of mean ± standard error of the mean (S.E.M.) presented are from representative experiments performed with triplicates for TL and duplicates for TA. Heatmap showing the means of pan-chromosomal (p.c.) and chromosome-specific (2p, 18p) TERRA in CRC cell lines relative to the expression level in SW480-LT. **B** In vitro analyses of isogenic SW480-LT and SW480 cells. Representative cell nuclei after telo-qFISH analyses are shown, size bar = 10 µm. Senescent cells were visualized by Senescence-Associated (SA) β-Galactosidase assay as blue stained cells 72 h after infection by adenovirus (AV) with multiplicity of infection (MOI) 10 for ectopic expression of TERTdn and eGFP as control, size bar = 100 µm. **C** Karyotypic constitution of a representative mitotic SW480-LT cell according to ISCN2020 is as follows: 103,XX,+X,+der(1)t(1;9)(q12;q11)x2,+2,+2,+2,+del(2)(p12),+der(2)t(2;11)(q36;q13)x2,+del(3)(q11),+del(3)(p21),+4,+4,+der(5)t(5;20)(q15;p12)x2,+7,+7,+der(7)add(7qter)x2,del(8)(q22),+der(8)add(8p),+der(8)t(8;10)(p11;q11),+del(9)(q32),+der(9)t(1;9)(q12;q11)x2,+11,+11,+11,+i(12p)x2,+13,+14,+14,+15,+15,+16,+16,+16,+17,+17,+del(18?)(q12?)x3,+der(19)add(19)(q13.3)x2,+20,+20,+der(20)t(5;20)(q15;p12)x3,+21,+21,+22,+mar1,mar2,+mar3,+mar4. This karyotype exhibits most of the described characteristic chromosome aberrations of the parental SW480 cells in two or more copies, indicating the same origin. STR profiling confirmed the identity with SW480. **D** In vitro growth of SW480-LT, SW480 and SW620. 2D colony formation, 3D growth in soft agar and cell invasion capacity of SW480, SW480-LT and SW620. Results of at least two independent experiments are shown. Bars represent Mean ± S.E.M. Unpaired t test was applied. *p* < 0.05 (*), *p* < 0.005 (**). **E, F** Tumor formation capacity of xenografted CRC models. Tumors were generated by subcutaneous injections of 5 × 10^6^ cells per model into the flanks of immunodeficient mice with replicates (*n* = 8). Tumor volume and weight at end points were measured and calculated as mean ± standard deviation values. Variation at different time points between the repeated measured tumor volumes was analyzed by two-way ANOVA with the Geisser-Greenhouse correction. Tumor weights per group were analyzed by *t* test. **E** Tumor volume and weight after 23 days, SW480: 940 ± 454 mm^3^, 0.889 ± 0.381 g; SW480-LT: 200 ± 152 mm^3^, 0.381 ± 0.317 g. **F** SW480 LV cell clones with ectopic TERRA (SW480-TERRA) or GFP (SW480-GFP) expression were infected with MOI 5 of AV TERTdn or AV GFP (control) 24 hours before transplantation. Tumor volume after 40 days for SW480-GFP and SW480-TERRA was 192 ± 179 mm^3^ and 62.6 ± 82.8 mm^3^, respectively. SW480-GFP and SW480-TERRA cell clones with TERTdn expression reached 44.6 ± 61.7 mm^3^ and 51.8 ± 62.5 mm^3^, respectively. Tumor weight after 40 days for SW480-GFP and SW480-TERRA cell clones treated with AV GFP or AV TERTdn was 0.508 ± 0.220 g, 0.222 ± 0.164 g, 0.143 ± 0.186 g and 0.180 ± 0.120 g, respectively

The isogenic SW480/SW480-LT cells were validated for their tumorigenicity in vivo when grown as xenotransplanted tumors in SCID mice (Fig. 2E). Tumor volumes increased faster for SW480 than for SW480-LT cells (*p* < 0.0001). After 23 days, the tumor weight variation was significant (*p* = 0.012) indicating that SW480-LT cells have less capacity to form tumors in mice. To further address whether the decreased tumor growth is related to increased TERRA expression in SW480-LT cells, we analyzed in vivo SW480 lentivirus (LV) cell clones available from a previous study that exogenously overexpress the telomeric part of TERRA or GFP under control of CMV promoter [22]. SW480 cell clones with ~1.6-fold elevated ectopic TERRA expression compared to controls with GFP showed reduced capacity to form tumors in mice (*p* = 0.0001) (Fig. 2F). A similar reduction in tumor growth was observed when the SW480 cell clones were pretreated with AV TERTdn compared to AV GFP as a control (*p* = 0.0001). SW480 cells expressing elevated levels of TERRA had a reduction in tumor weight similar to cells with TERTdn expression (*p* < 0.05).

In conclusion, this study investigated the clinical impact of telomere and proliferation associated parameters on human CRC patients and evaluated the function of telomere transcripts on in vivo tumor growth. The combination of high TERRA and low PCNA expression in T compared to adjacent N tissues was associated with favorable CRC patient prognosis. In addition, high TERRA expression in telomerase positive CRC cells xenotransplanted in SCID mice suppressed tumor growth and therefore supported the clinical finding.

Our observations are in line with several studies that report the association of high TERRA expression with favorable patient survival (reviewed in [23]). Another translational study of 60 CRC patients revealed that 18p TERRA, in conjunction with CEA, served as a prognostic factor, particularly influencing progression-free survival and, to a lesser extent, OS [12]. In a cohort of 46 astrocytoma patients, TERRA was associated with favorable patient prognosis [24]. Moreover, reduced TERRA expression in the head and neck squamous cell carcinomas was linked to more aggressive tumor growth and an unfavorable patient prognosis [25]. Recent studies in several other cancers, including hepatocellular carcinoma, endometrial carcinogenesis, acute myeloid leukemia and cutaneous T-cell lymphoma, support a potential tumor suppressive role of TERRA [26–30]. In contrast, TERRA levels were not significantly different between tumor samples and their matched non-neoplastic controls when TERRA was estimated by RNA-seq in another study, which included both tissue and blood samples [31]. A previous study has shown that TERRA interacts with TERC and TERT, thereby regulating telomerase activity [6]. In line with this, we found a negative correlation between p.c.TERRA as well as 2p and 18p TERRA with TA. Furthermore, in CRC tumor tissue a positive correlation between TERRA and TL suggests an influence of TERRA on chromosome stability.

As expected, TA, TERT as well as TERC were higher in T than in N tissue which is in line with previously published reports on mRNA and protein level [32–35]. We identified PCNA as factor that correlates positively with TERRA in the studied cohort. SILAC-based RNA–protein interaction analysis and affinity purification did not detect PCNA as an UUAGGG-associated protein [36, 37]. This leads us to speculate that PCNA might be regulated indirectly via yet unidentified mediators or, alternatively, without a direct causal link acting independently.

We observed a positive correlation between TERRA and ESRPs, implying a potential role for alternative splicing in the regulation of TERRA. This association may have implications for both telomere biology and EMT. The impressive correlation between TERRA, ESRP, and PCNA identified in T but not N tissue may have implications for CRC biology and targeting. Transcription factors such as ZEB1 and Snail1 are regulators of TERRA and EMT [18, 19], and together with PCNA phosphorylation may be involved in regulating this interplay [38]. However, the mechanisms behind these correlations need to be investigated in detail at the cellular and spatial level in the CRC tissues. The relatively small sample size of 68 patients in our study presents several limitations. The reduced power increases the likelihood of type II errors, where true effects may go undetected. This limitation also results in wider confidence intervals, indicating less precise estimates. Furthermore, there is an increased heightened risk of bias if the sample is not representative of the broader population. Consequently, the generalizability of our findings may be limited. Further independent studies with larger patient cohorts are necessary to corroborate the association between PCNA protein expression in combination with TERRA expression and clinical outcome. Furthermore, in vitro studies should be performed to test a possible functional relationship between PCNA, ESRPs and TERRA. The reduced tumor growth upon TERRA overexpression in vivo suggests that TERRA acts as a tumor suppressor in CRC and may be a tool to target telomerase positive CRC. Several mechanisms are possible and need to be evaluated in the future. First, data of this study indicate that CRC cells are addicted to TA, and TERRA blocks TA, as does expression of dnTERT, causing replicative senescence [22]. Second, TERRA are a class of transcripts with G-quadruplex structure known to trigger the innate immune response [39] with extrinsic and systemic function of TERRA [40]. Third, whether constitutive expression of TERRA could induce chromosomal perturbations leading to chromosome instability, and thereby hindering CRC proliferation needs to be investigated.

## Electronic supplementary material

Below is the link to the electronic supplementary material.

Supplementary Material 1

Supplementary Material 2

## Data Availability

No datasets were generated or analysed during the current study.
