# Supplementary material for: Telomere transcripts act as tumor suppressor and are associated with favorable prognosis in colorectal cancer with low proliferating cell nuclear antigen expression
Source: Cell Oncol (Dordr). 2024 Sep 2;48(1):239–47. doi: 10.1007/s13402-024-00986-y (PMC11850466; doi:10.1007/s13402-024-00986-y)
Supplement: Supplementary file 1 — Supplementary Material 1 [file 13402_2024_986_MOESM1_ESM.docx]

**Supplementary Material and Methods: Kienzl et al.**

**1. Patients and human colorectal cancer samples**

For this study, clinical data as well as tissue samples were identical to a previously published patient cohort [[1](#_ENREF_1)]. Primary CRC tissue samples of 68 patients were obtained from tumor resections at Clinic Favoriten, until 2020 known as Kaiser Franz Josef (KFJ) Hospital, Kundratstraße 3, 1100 Vienna, Austria after informed consent and with approval from the local Ethics Committee (Municipal Department of Vienna MA15, ethics numbers EK 06-150-VK and EK-05-004-VK). Tumor (T) and from the resection border matched adjacent non-neoplastic colorectal epithelium as non-tumor (N) tissue, were rapidly frozen in liquid nitrogen immediately after surgical removal and stored at -80°C. Demographic, surgical, pathological and clinical follow-up data for all patients were collected until end of 2013. Overall survival (OS) was calculated from the date of the initial surgery, confirming CRC histologically, until the time of death or until the last follow up date.

**2. Cell lines and mice**

CRC cell lines Caco-2 (HTB-37), HCT116 (CCL-247), HT-29 (HTB-38), SW480 (CCL-228), SW620 (CCL-227) were obtained from the American Type Culture Collection (ATCC, Manassas, VA). Cells were cultured with recommended medium, except for SW480, SW480-LT and SW620, which were cultured with RPMI-1640 (Sigma-Aldrich, St. Louis, USA) for comparable growth conditions. Colorectal adenoma cell lines LT97 and Vaco235 were established and cultured as described [[2](#_ENREF_2), [3](#_ENREF_3)]. SW480 with ectopic overexpression of TERRA and GFP were described [[4](#_ENREF_4)]. All cell lines were grown in medium supplemented with 10% fetal calf serum (FCS) and 1% Penicillin/Streptomycin under standard tissue culture conditions (5% CO2 at 37 °C) and authenticated by short tandem repeat (STR) profiling (Eurofins Genomics, Vienna, Austria).

Immune deficient female SCID (C.B-17/IcrHsd-Prkcdscid) mice were used for xenotransplantation of human CRC cell lines maintained according to the Austrian guidelines for animal care and protection. 5 x10^6^ CRC cells were subcutaneously injected into the flanks of mice. Tumor growth was monitored by estimating the tumor volume as described [[5](#_ENREF_5)].

**3. Cell culture assays**

Cell morphology was visualized by phase-contrast microscope (Eclipse Ti, Nikon instruments, NY, USA). Cell number and cell size profile were measured with an automated cell counter (Casy TTC, OLS). For cell growth analyses, cell lines were seeded at a density of 100,000 cells in 60x15 mm Petri dishes. Cells were counted after 24 h intervals for 4 days and cell growth rates were calculated as population doubling time (PDT) by nonlinear regression with 95% CI.

For the 2D colony formation assay, 100 cells per well were seeded in triplicate on 6-well plates and 3 ml growth medium supplemented with 10% FCS. After 24 h growth the medium was aspirated and changed. Nine days afterwards the medium was removed and replaced with ice cold methanol and incubated overnight at -20°C. Cells were washed with PBS and stained with 0.01% crystal violet solution as described [[6](#_ENREF_6)]. To assess colony formation the Lucia Morphometry Software (Laboratory Imaging, Praha, CZ) was applied.

The 3D soft agar growth assay was done in triplicate as described [[7](#_ENREF_7)]. In detail, 5 x 1000 cells were suspended in RPMI-1640 medium containing 20% FCS including 0.3% agar (Sigma) and were seeded onto 6-well plates, with RPMI-1640 supplemented with 20% fetal calf serum and 0.6% agar. After 2-4 weeks the cell growth was inspected by microscope (Eclipse Ti, Nikon instruments, NY, USA) and colonies bigger than 100 µm with around more than 10 cells were counted at five areas per well and the average colony number was calculated.

Cell migration and invasion assay was performed in 24-well plates with 800µl of medium per well and transparent PET membrane inserts with 8.0µm pore sizes (BD Falcon). 20,000 cells in 200µl medium were added in the upper compartment of the filter. Three days later, the filter was removed, and the cells that migrated through the pore and fell to the bottom of the well were allowed to grow for another 4 days. Then the cells were stained and analyzed as described for colony formation assay.

Cell senescence assay and inhibition of TA by recombinant adenovirus (AV) expressing dominant-negative (dn) TERT and control AV with eGFP expression was performed with indicated multiplicity of infection (MOI) as described [[4](#_ENREF_4)].

**4. Karyotype analyses with inverted DAPI banding**

Logarithmically growing cell cultures were exposed to colcemid (0.1μg/ml) (Gibco) for 1 to 2 hours, at 37°C in 5% CO2. Cells were harvested by trypsinization (Gibco), suspended in medium, and spun down (10 minutes at 1500 rpm). Supernatant was removed completely, and 5 ml of 0.075 M KCl (Sigma) at room temperature was added drop by drop. The cells were incubated for 20 minutes at room temperature, and then 1 ml of fixative [3× methanol (AppliChem GmbH, Darmstadt, Germany)/1× CH_3_COOH (Merck, Darmstadt, Germany)] was added. Cells were spun down (10 minutes at 1500 rpm), supernatant was removed, fixative was added, and the cells were re centrifuged for 10 minutes at 1500 rpm. Finally, cells were dropped onto wet microscope slides and left to air-dry. For inverted DAPI staining, slides were counterstained and mounted with 0.1μg/ml DAPI in VECTASHIELD antifade medium (Vector Laboratories, Burlingame, CA). Cytogenetic analyses were performed using a 63x magnification lens on a fluorescent Axio-Imager Z1, Zeiss microscope, equipped with a MetaSystems charge-coupled device camera and the MetaSystems Isis software. As representatives, the karyotypes of the major clone per cell harvest were considered and at least 25 metaphases stained with inverted DAPI banding from one harvest were completely analyzed. Because of the complexity of chromosomal rearrangements, karyotypes were written in the extended form according to International System for Human Cytogenomic Nomenclature (ISCN) 2020 [[8](#_ENREF_8)].

**5. Telomere length and telomerase activity**

DNA was isolated as described [[4](#_ENREF_4), [9](#_ENREF_9)] and the relative telomere length (relTL) was measured as T/S relative quantity (RQ) value by using a monochrome multiplex quantitative PCR (qPCR) method [[10](#_ENREF_10)] with 36B4 as single (S) copy reference gene [[11](#_ENREF_11)]. In brief, 20 ng DNA aliquots were set up in 8-µl volume using iQ SYBR Green Supermix with 200nM of each oligonucleotide and MyiQ Single Color Real-Time PCR Detection System (Bio-Rad, Austria). Absolute TL analyses for 20 CRC cases was performed with 1 μg DNA by terminal restriction fragment (TRF) analyses to determine the mean TL in kilobase pairs as described [[9](#_ENREF_9)]. Mean TL values and TL RQ values were plotted, and a standard curve was fitted. From this curve, absolute TL was extrapolated for all other samples where TL RQ was available. Telo-qFISH analyses for TL detection in single cells on interphase nuclei was performed using Telomere PNA FISH kit/FITC (Dako Denmark A/S, Glostrup, Denmark) as described [[12](#_ENREF_12)].

Telomerase activity (TA) was quantified as total product generated (TPG) units of protein extracts by qPCR-telomeric repeat amplification protocol (TRAP) [[4](#_ENREF_4)] and tissue samples from 38 of 68 cases (56%) were validated by polyacrylamide gel electrophoresis (PAGE)-TRAP [[9](#_ENREF_9)].

**6. Transcript quantification**

RNA extraction from tissue and cells as well as reverse transcription (RT) and real-time monochrome singleplex quantitative polymerase chain reaction (qPCR) were performed as described previously [[1](#_ENREF_1), [9](#_ENREF_9)]. Briefly, tissue was homogenized using ceramic beads with Precellys tissue homogenizer (Peqlab, Germany) and TRIzol reagent (Invitrogen, USA). RNA was isolated according to TRIzol manufacturer’s instructions. RNA concentration and purity were measured with NanoDrop 1000 spectrophotometer (Thermo Fisher Scientific, USA). One µg RNA was reverse transcribed using RevertAid First Strand complementary DNA (cDNA) synthesis kit and hexanucleotide primers with RevertAid Premium Reverse Transcriptase (Fermentas, Germany) at 55°C as described by the manufacturer. The elevated temperature ensures quantitative polymerase reactions of complex sequence structures such as for TERRA transcripts [[13](#_ENREF_13)]. PCR was carried out in triplicate on cDNA aliquots with GoTaq qPCR Master Mix (Promega, USA) and 200 nM primers each, using Applied Biosystems 7500 Fast Real-time qPCR System (Applied Biosystems, Foster City, USA). Amplification of possible DNA contaminations were randomly tested by qPCR of cDNA reactions without RT enzyme. Cycle threshold (Ct) quantities found were similar to those of non-template controls and negligible for quantification of transcript levels. RQ values were calculated by the Delta Delta Ct method [[14](#_ENREF_14)]. In brief, cycle threshold (Ct) values of genes were normalized to those of 36B4 reference gene as well as to those of gene expression of an arbitrary reference sample. Efficiencies of qPCRs were determined by standard curves generated as 1:2 serial dilutions of amplicons and used for calculating RQ values [[15](#_ENREF_15)]. Primer sequences and efficiencies are outlined in **Supplementary Table 3**.

**7. Statistical analysis**

For analysis of TRF blots Image Lab software version 6.0 (Bio-Rad Laboratories Inc.) was used. PDT was determined by GraphPad Prism software version 8 with standard nonlinear regression analyses of exponential growth equation and calculation of 95% confidence intervals (CI) using the more accurate asymmetrical CI. Interpretation, statistical analysis and graphical illustration of data were performed using Seaborn python library [[16](#_ENREF_16)] and R packages “corrplot” [[17](#_ENREF_17)] and “PerformanceAnalytics”. Survival curves were generated using R version 4.0.4 with packages “survival” [[18](#_ENREF_18)] and “survminer” and equality of the survivor function across groups was tested using log rank method. The Cox regression model was employed for both univariate and multivariate survival analysis. Log-transformed RQ values were utilized, and for multivariate analysis, the model was adjusted for age, stage (dichotomized into I-II and III-IV), grade, site, gender, and microsatellite status. All statistical tests were two-sided and a P value ≤ 0.05 was considered significant. For correlation analysis, RQ values were log transformed and linear regression using Pearson correlation was performed. A paired t-test was employed after confirming the normal distribution of the data to assess differences between two groups. Low, moderate, high and very high significant differences were marked with * (P value < 0.05), ** (P value < 0.01), *** (P value < 0.001) and **** (P value < 0.0001), respectively.

**8. Supplementary Tables and Figures**

**Supplementary Table 1:** Clinical characteristics and telomere-related data of CRC patient cohort.

**Supplementary Table 2:** Demographic characteristics comparison of CRC patients grouped by median according to TERRA and PCNA expression levels.

**Supplementary Table 3:** List of oligonucleotides used for qPCR assays and efficiencies for quantification.

**Supplementary Figure 1.** TERRA expression in CRC cases present (TA pos; n=61) or absent (TA neg, n=7) for detected TA in tumor. RQ levels from pan-chromosomal TERRA in tumor and non-tumor tissues were analyzed. Results from Mann-Whitney test are shown. p > 0.05 not significant (ns), p < 0.005 (**).

**Supplementary Figure 2.** TERRA transcription from different chromosome ends in tumor (T) and adjacent non-tumor (N) tissue of CRC cases. A subset of clinical samples (n=5) was analyzed with all TERRA assays shown in Figure 2A. Compared to others, the results show that 2p and 18p are most similar to p.c.TERRA expression in T tissue and as a T/N ratio. In addition, TERRA expression from different chromosome ends varies in the tissues examined.

**Supplementary Figure 3.** Expression of proliferation associated genes in tumor (T) and adjacent non-tumor (N) tissue of CRC cases. (A) Three CRC cases each were grouped according to low (green), middle (blue) and high (red) pan-chromosomal TERRA transcript levels in T tissue. Scatter plots of TERRA and proliferation associated genes (MKI67, MYC, CCND1, CCNA2 and PCNA) are presented in T and N tissue as well as T/N ratio. A linear regression line was fitted and Pearson’s r as well as p value are shown.

**Supplementary Figure 4.** Overall survival rates of CRC patients by Kaplan-Meier analyses depend on telomerase activity (TA), PCNA and TERRA expression. (A) Survival curves of patients grouped into four combinational categories by low and high T/N expression ratios of TA and PCNA as well as (B) of TA and p.c.TERRA above and below the median. The median of each variable was selected as the cut-off value. The survival curves are presented with pointwise confidence bands for smoothed hazard functions. The x-axis represents time in months.

**Supplementary Figure 5.** Telomere restriction fragment (TRF) analyses of CRC cell models (n=7). (A) Telomeres detected by hybridization with a radio-labeled probe and mean TL in kbp was calculated as mean TRF size by comparison with molecular weight marker (MWM) as described , SAOS-2 are a osteosarcoma cell model with long telomeres and used as control [[9](#_ENREF_9)]. (B) DNA stained on agarose gel with ethidium bromide before TRF analysis to proof efficient restriction digestion.

**Supplementary Figure 6:** In vitro growth of CRC cell models SW480-LT and SW480. (A) Cell proliferation kinetics. Each point of the growth kinetics represents the mean of two independent experiments each with technical quadruplicates, with cell numbers assessed by automated cell counter. (B) Outgrowth experiment of SW480-LT cell clone tagged by GFP and mixed with SW480. GFP positive cells were measured after one passage of co-culture. Bars represent Mean ± S.E.M. of two independent experiments. (C) Cell morphology of subconfluent SW480-LT and SW480 with higher density. Scale bar represent 100 µm. (D) Mean cell size diameters were measured by Casy cell counter in µm. Representative results of at least two independent experiments are shown. Bars represent Mean ± S.E.M.

**Supplementary Figure 7:** Tumor weights of the surgically removed xenotransplanted tumors were measured with a balance after the last day and are summarized by box graph with min to max whiskers. Tumor weights per group were analyzed by unpaired t test (A) or by Mann-Whitney test (B) depending whether there was a normal distribution. (A) SW480 and SW480-LT cells. (B) SW480 LV cell clones with ectopic TERRA (SW480-TERRA) or GFP (SW480-GFP) expression infected with MOI 5 of AV TERTdn or AV GFP (control).

**9. Supplementary References**

1 A.J. Deloria, D. Höflmayer, P. Kienzl, J. Łopatecka, S. Sampl, M. Klimpfinger, T. Braunschmid, F. Bastian, L. Lu, B. Marian, S. Stättner and K. Holzmann, Oncotarget 7, 73800-73816 (2016) doi: 10.18632/oncotarget.12070

2 M. Richter, D. Jurek, F. Wrba, K. Kaserer, G. Wurzer, J. Karner-Hanusch and B. Marian, Eur J Cancer 38, 1937-1945 (2002) doi: 10.1016/s0959-8049(02)00158-2

3 J.K. Willson, G.N. Bittner, T.D. Oberley, L.F. Meisner and J.L. Weese, Cancer Res 47, 2704-2713 (1987)

4 T. Kreilmeier, D. Mejri, M. Hauck, M. Kleiter and K. Holzmann, Genes (Basel) 7, 46 (2016) doi: 10.3390/genes7080046

5 G. Zulehner, M. Mikula, D. Schneller, F. van Zijl, H. Huber, W. Sieghart, B. Grasl-Kraupp, T. Waldhör, M. Peck-Radosavljevic, H. Beug and W. Mikulits, Am J Pathol 176, 472-481 (2010) doi: 10.2353/ajpath.2010.090300

6 C. Heinzle, A. Gsur, M. Hunjadi, Z. Erdem, C. Gauglhofer, S. Stättner, J. Karner, M. Klimpfinger, F. Wrba, A. Reti, B. Hegedus, A. Baierl, B. Grasl-Kraupp, K. Holzmann, M. Grusch, W. Berger and B. Marian, Cancer Res 72, 5767-5777 (2012) doi: 10.1158/0008-5472.can-11-3654

7 C. Gauglhofer, J. Paur, W.C. Schrottmaier, B. Wingelhofer, D. Huber, I. Naegelen, C. Pirker, T. Mohr, C. Heinzle, K. Holzmann, B. Marian, R. Schulte-Hermann, W. Berger, G. Krupitza, M. Grusch and B. Grasl-Kraupp, Carcinogenesis 35, 2331-2338 (2014) doi: 10.1093/carcin/bgu151

8 I.S.C.o.H.C. Nomenclature, J. McGowan-Jordan, R.J. Hastings and S. Moore, ISCN 2020: An International System for Human Cytogenomic Nomenclature (2020), (Karger, 2020).

9 S. Sampl, S. Pramhas, C. Stern, M. Preusser, C. Marosi and K. Holzmann, Transl Oncol 5, 56-65 (2012) https://doi.org/10.1593/tlo.11202

10 R.M. Cawthon, Nucleic Acids Res 37, e21 (2009) doi: 10.1093/nar/gkn1027

11 G. Wultsch, T. Setayesh, M. Kundi, H. Al-Serori, T. Kreilmeier-Berger, N. Ropek, K. Holzmann, A. Nersesyan and S. Knasmüller, Mutat Res Genet Toxicol Environ Mutagen 836, 78-81 (2018) doi: 10.1016/j.mrgentox.2018.05.004

12 T. Kreilmeier, S. Sampl, A.J. Deloria, I. Walter, M. Reifinger, M. Hauck, L.B. Borst, K. Holzmann and M. Kleiter, Mol Carcinog 56, 923-935 (2017) doi: 10.1002/mc.22546

13 A. Porro, S. Feuerhahn, P. Reichenbach and J. Lingner, Mol Cell Biol 30, 4808-4817 (2010) doi: 10.1128/mcb.00460-10

14 K.J. Livak and T.D. Schmittgen, Methods 25, 402-408 (2001) doi: 10.1006/meth.2001.1262

15 M.W. Pfaffl, Nucleic Acids Res 29, e45 (2001) doi: 10.1093/nar/29.9.e45

16 M.L. Waskom, Journal of Open Source Software 6, 3021 (2021) doi: 10.21105/joss.03021

17 T. Wei and V. Simko, R package 'corrplot': Visualization of a Correlation Matrix, 2021.

18 M.T. Terry and M.G. Patricia, Modeling Survival Data: Extending the Cox Model, (Springer, New York, 2000).

19 R.M. Cawthon, Nucleic Acids Res 30, e47 (2002) doi: 10.1093/nar/30.10.e47

20 P. Potemski, E. Pluciennik, A.K. Bednarek, R. Kusinska, R. Kubiak, D. Jesionek-Kupnicka, C. Watala and R. Kordek, Pathology, research and practice 202, 491-495 (2006) doi: 10.1016/j.prp.2006.02.005

21 H.S. Christensen, A. Daher, K.J. Soye, L.B. Frankel, M.R. Alexander, S. Laine, S. Bannwarth, C.L. Ong, S.W. Chung, S.M. Campbell, D.F. Purcell and A. Gatignol, Journal of virology 81, 5121-5131 (2007) doi: 10.1128/JVI.01511-06

22 J.S. Dome, C.A. Bockhold, S.M. Li, S.D. Baker, D.M. Green, E.J. Perlman, D.A. Hill and N.E. Breslow, Journal of clinical oncology : official journal of the American Society of Clinical Oncology 23, 9138-9145 (2005) doi: 10.1200/JCO.2005.00.562

23 N.W. Kim and F. Wu, Nucleic Acids Res 25, 2595-2597 (1997) doi: 10.1093/nar/25.13.2595

24 N. O'Callaghan, V. Dhillon, P. Thomas and M. Fenech, BioTechniques 44, 807-809 (2008) doi: 10.2144/000112761

25 C.M. Azzalin, P. Reichenbach, L. Khoriauli, E. Giulotto and J. Lingner, Science 318, 798-801 (2007) doi: 10.1126/science.1147182
