# Supplementary material for: Telomere transcripts act as tumor suppressor and are associated with favorable prognosis in colorectal cancer with low proliferating cell nuclear antigen expression
Source: Cell Oncol (Dordr). 2024 Sep 2;48(1):239–47. doi: 10.1007/s13402-024-00986-y (PMC11850466; doi:10.1007/s13402-024-00986-y)
Supplement: Supplementary file 2 — Supplementary Material 2 [file 13402_2024_986_MOESM2_ESM.pdf]

Supplementary Figure 1

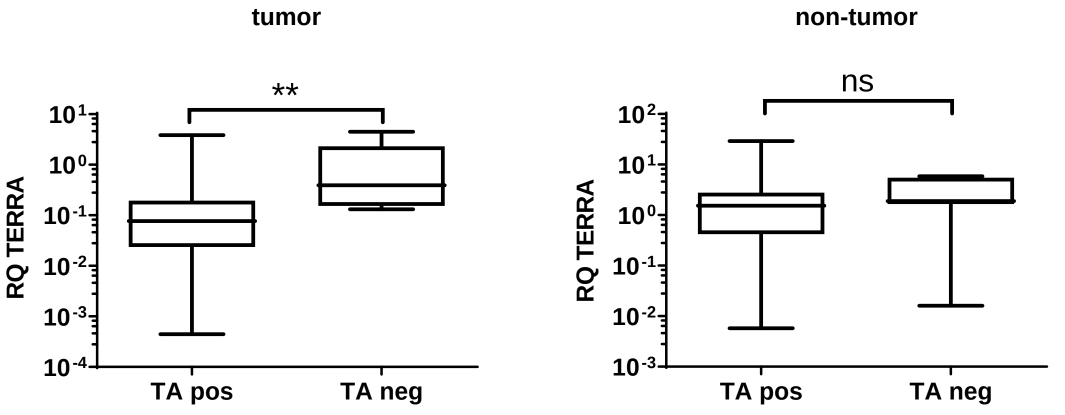

Supplementary Figure 2

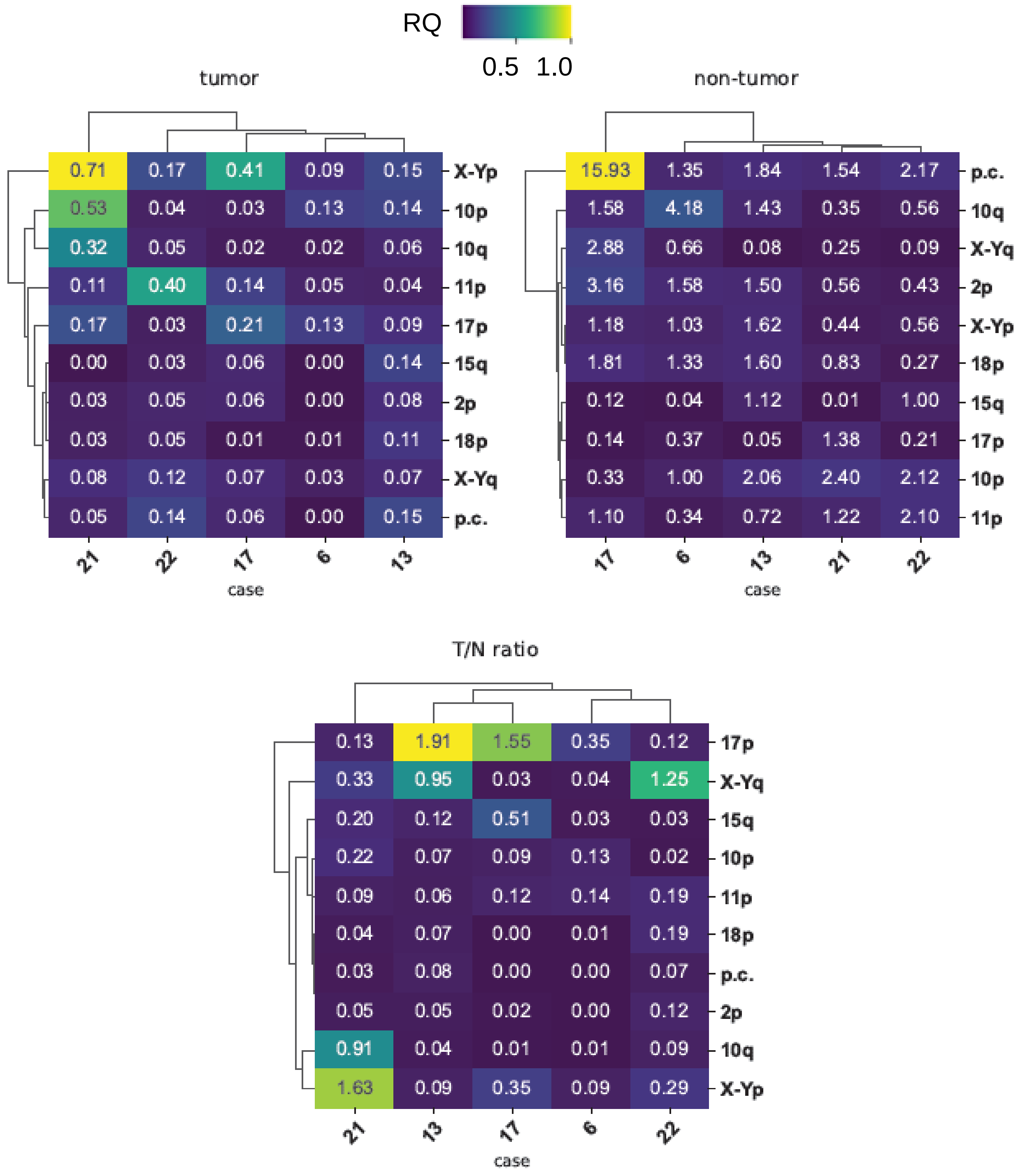

Supplementary Figure 3

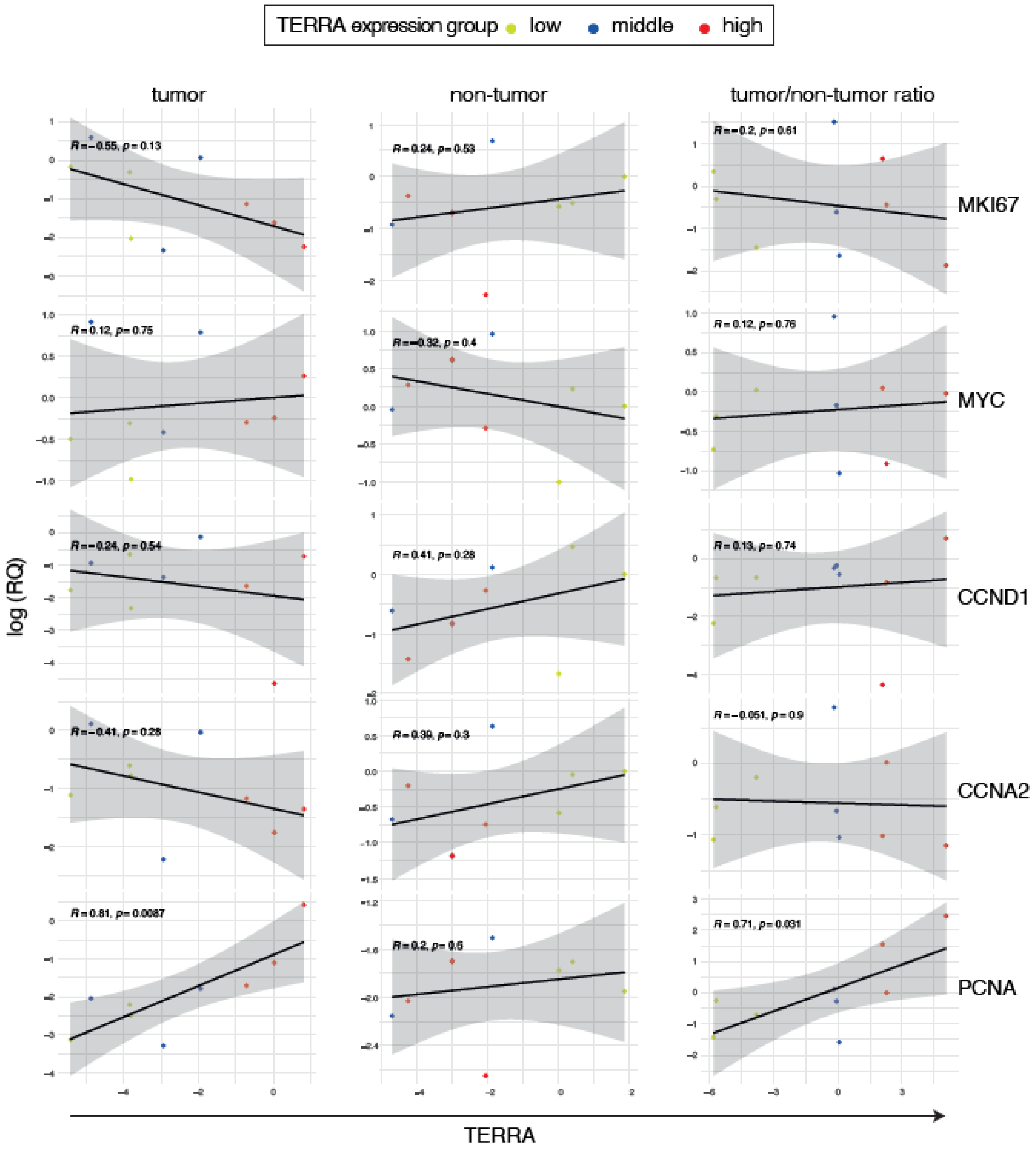

Supplementary Figure 4

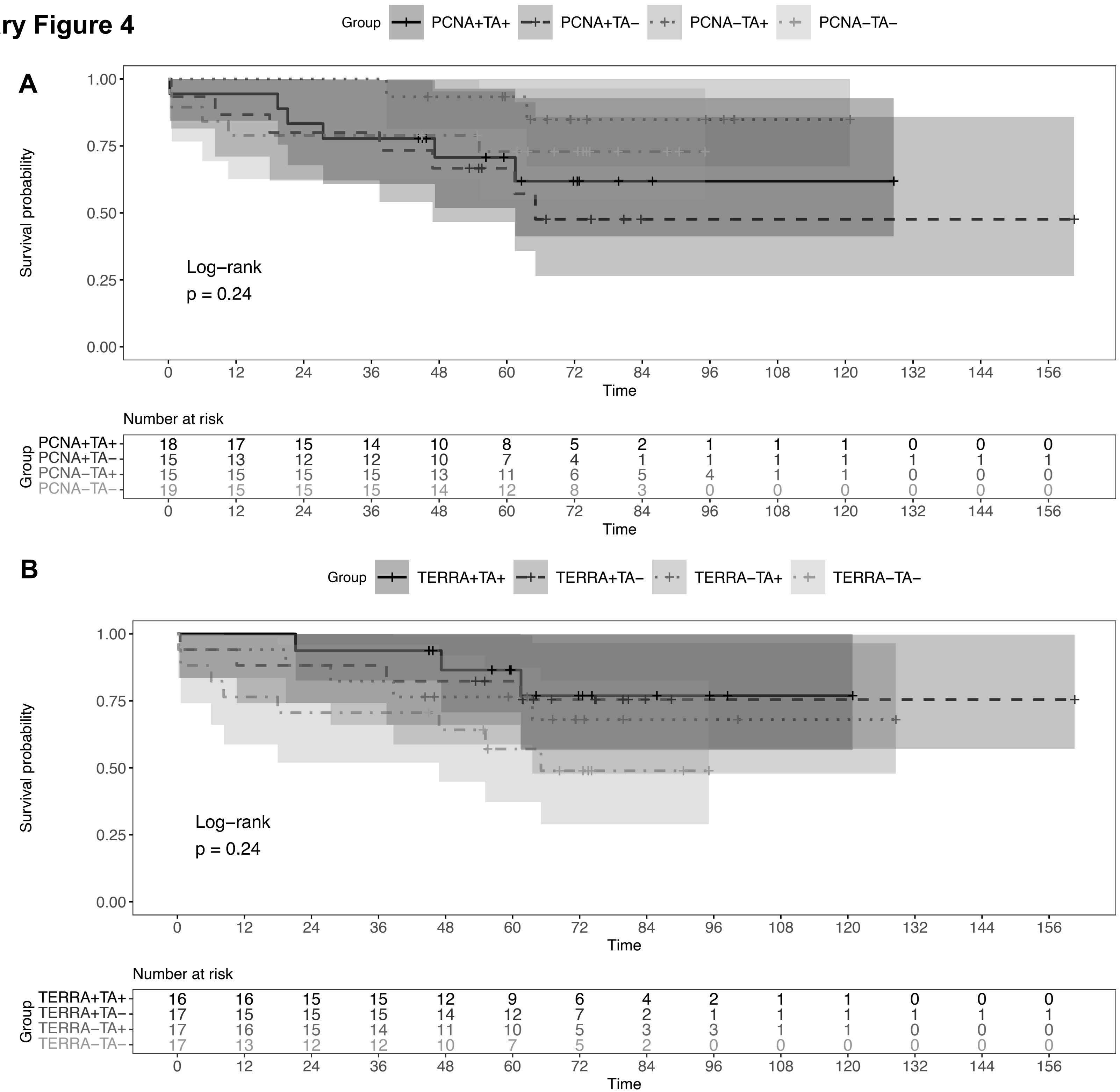

Supplementary Figure 5

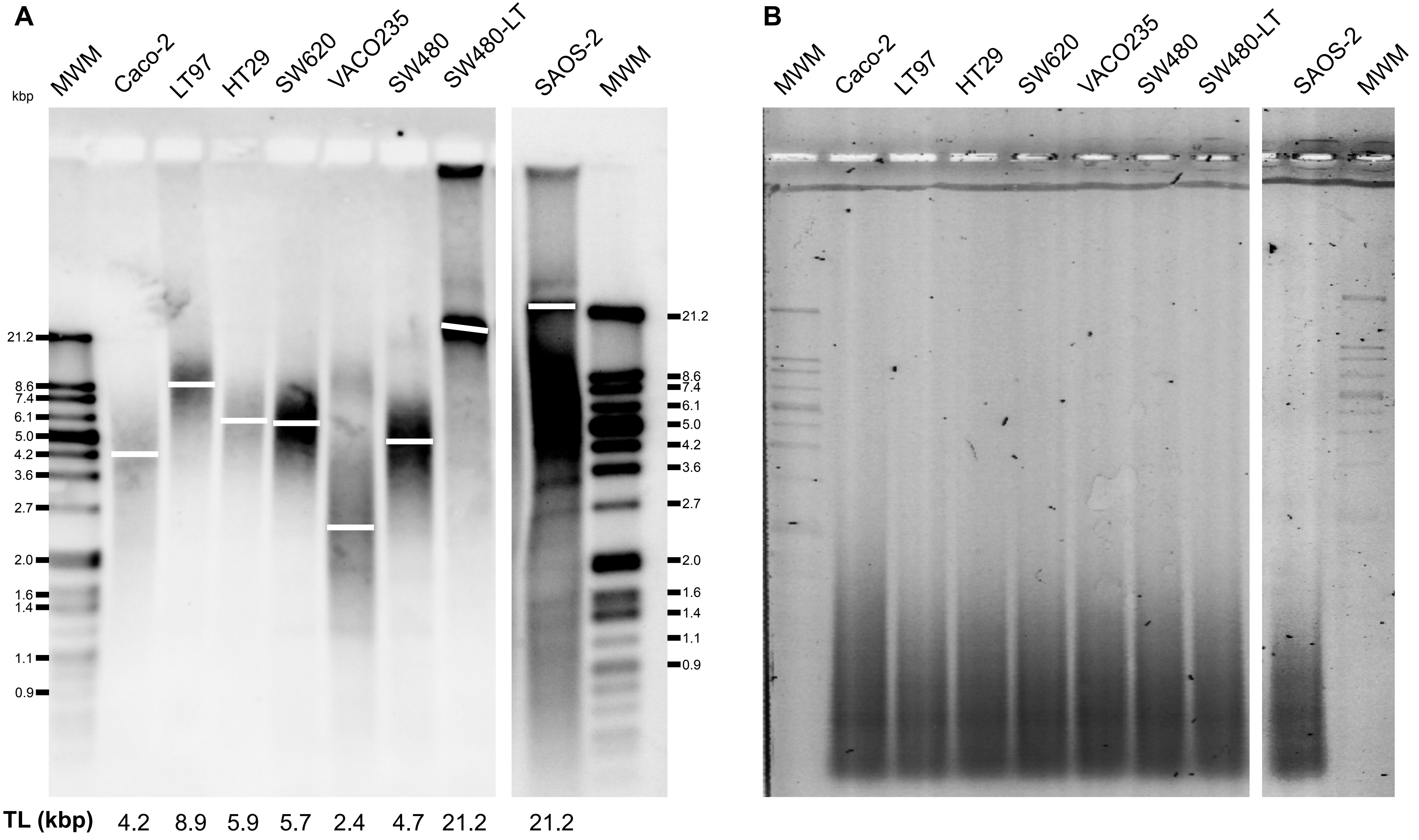

Supplementary Figure 6

A

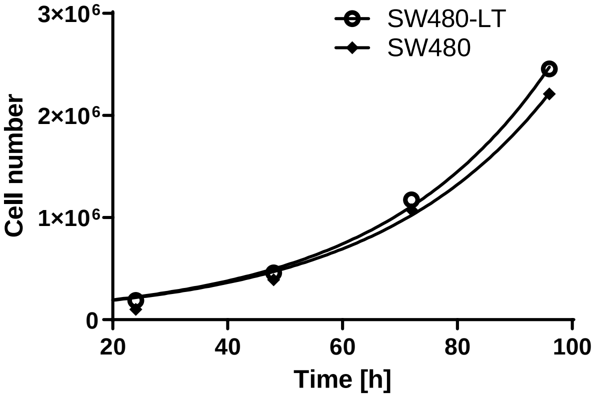

	PDT [h]	95% CI [h]
SW480-LT	20.7	20.6 to 20.9
SW480	21.5	21.1 to 21.8

B

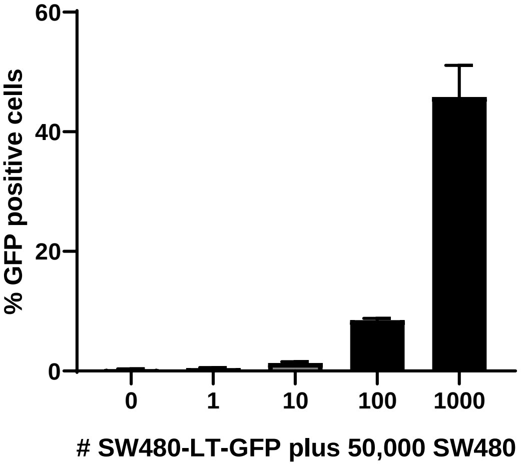

C

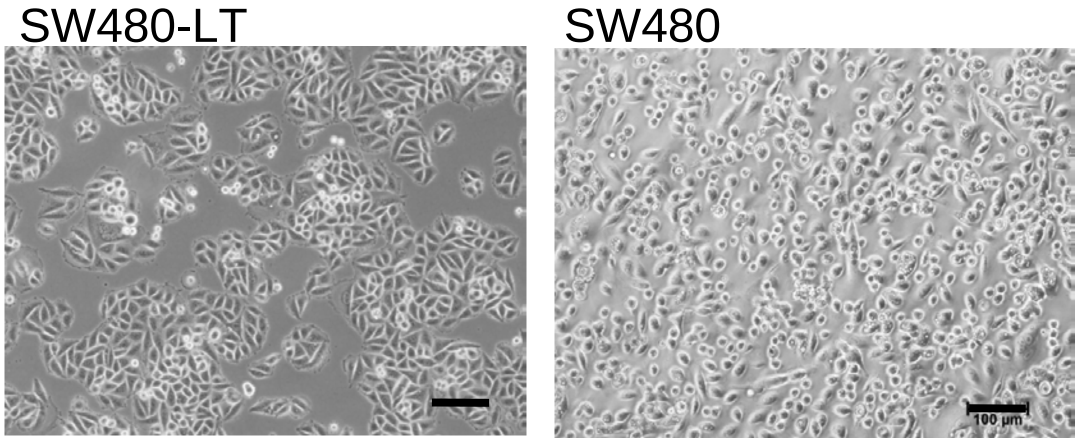

D

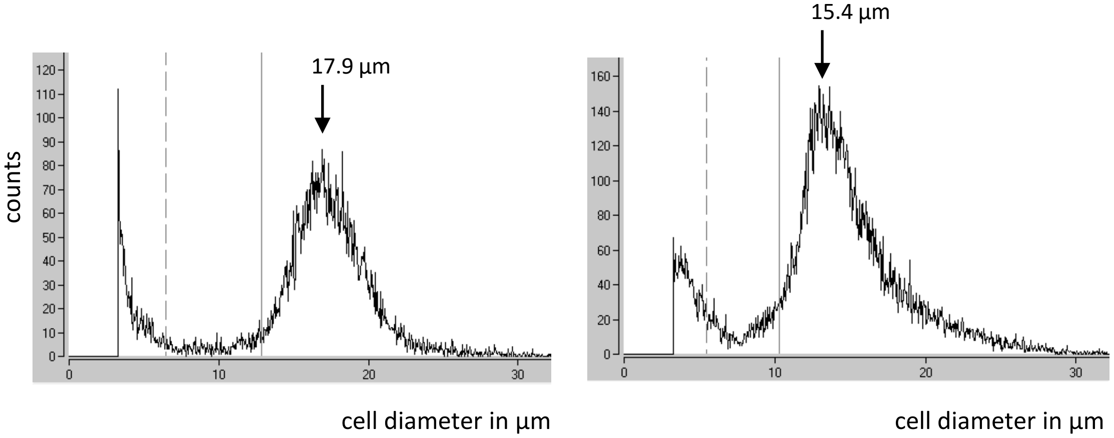

Supplementary Figure 7

A

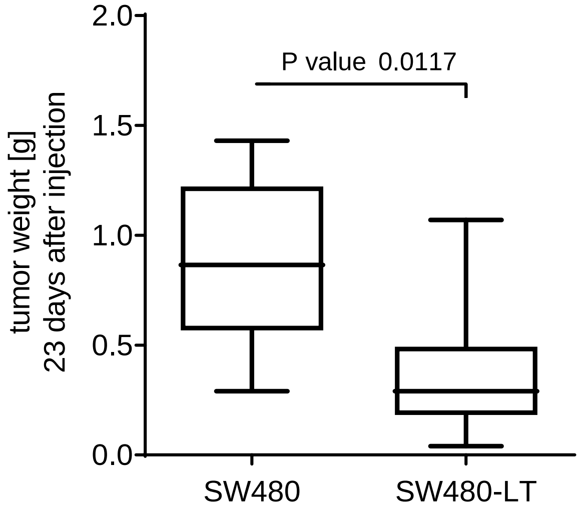

B

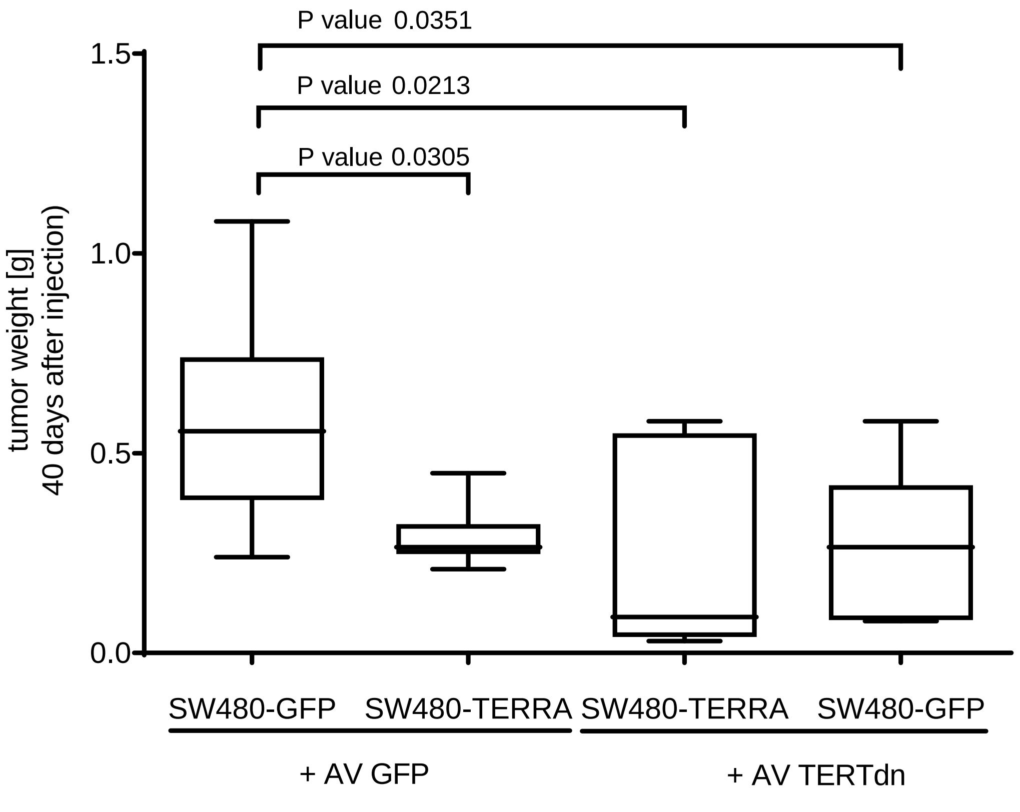

Supplementary Table 1. Clinical characteristics and telomere-related data of CRC patient cohort.

Number (n) of patients	n	68
Age in years	Median (Range)	70 (26 - 87)
Gender, n (%)	Female	26 of 68 (38.2)
	Male	42 of 68 (61.8)
UICC tumor stage, n (%)	I	11 of 68 (16.2)
	II	28 of 68 (41.2)
	III	21 of 68 (30.9)
	IV	7 of 68 (10.3)
	n.a.	1 of 68 (1.5)
WHO grade, n (%)	2	45 of 68 (66.2)
	3	22 of 68 (32.4)
	n.a.	1 of 68 (1.5)
Site, n (%)	colon	55 of 68 (80.9)
	rectum	13 of 68 (19.1)
Microsatellite instability, n (%)	stable	57 of 68 (83.8)
	instable	11 of 68 (16.2)
Metastasis, n (%)	yes	7 of 68 (10.3)
	no	60 of 68 (88.2)
	n.a.	1 of 68 (1.5)
RQ T<N, n (%)	p.c. TERRA	61 of 67 (91.0)
	2p TERRA	61 of 67 (91.0)
	18p TERRA	59 of 67 (88.1)
	TERT	20 of 66 (30.3)
	TERC	18 of 66 (27.3)
kpb T<N, n (%)	TL	24 of 67 (35.8)
TPG units T<N, n (%)	TA	3 of 67 (4.5)

Supplementary Table 2. Demographic characteristics comparison of CRC patients grouped by median according to TERRA and PCNA expression levels. +, patients with values higher than median; - patients with values lower than median.

	TERRA+ PCNA- (n = 13)	TERRA- PCNA+ (n = 13)	P value*
**age (years), median (range)**	68 (57-82)	73 (26-81)	0.86
**Tumor stage, n (%)**			0.48
I	3 (23.1)	1 (7.6)	
II	5 (38.5)	6 (46.2)	
III	4 (30.8)	6 (46.2)	
IV	1 (7.6)	0 (0)	
**Grade, n (%)**			0.69
2	9 (69.2)	7 (53.8)	
3	4 (30.8)	6 (46.2)	
**Site, n (%)**			1.00
colon	11 (84.6)	10 (76.9)	
rectum	2 (15.4)	3 (23.1)	
**Microsatellite instability, n (%)**			0.59
stable	10 (76.9)	12 (92.4)	
instable	3 (23.1)	1 (7.6)	
**gender, n (%)**			0.11
female	8 (61.5)	3 (23.1)	
male	5 (38.5)	10 (76.9)	

* Wilcoxon test was used to compare groups for age and the Chi-square test for categorical variables.

Supplement Table 3. List of oligonucleotides used for qPCR assays and efficiencies for quantification.

Primer	Sequenz (5´- 3´)	Efficiency	Reference
**Gene expression**			
36B4-forward	CCCATTCTATCATCAACGGGTACAA	0.94	[19]
36B4-reverse	CAGCAAGTGGGAAGGTGTAATCC		
PCNA-forward	GAGGAGGAAGCTGTTACCATAGAG	0.91	This study
PCNA-reverse	CTCGATCTTGGGAGCCAAGTAG		
CCNA2-forward	CTACACAGTCACGGGACAAAG	0.96	This study
CCNA2-reverse	GGTGAAGGTCCATGAGACAAG		
CCND1-forward	ATCAAGTGTGACCCGGACTG	0.83	This study
CCND1-reverse	ACCTCCTCCTCCTCCTCTTC		
MKI67-forward	TCCTTTGGTGGGCACCTAAGACCTG	1.01	[20]
MKI67-reverse	TGATGGTTG AGGTCGTTCCTTGATG		
MYC-forward	GCTCCTGGCAAAAGGTCAGAGTCTGG	0.98	[21]
MYC-reverse	GGGGCTGGTTGCATTTTCGGTTGTTGC		
TERC-forward	TCTAACCCTAACTGAGAAGGGGCG	0.85	This study
TERC-reverse	GTTTGCTCTAGAATGAACGGTGGAAG		
TERT-forward	CGGAAGAGTGTCTGGAGCAA	0.83	[22]
TERT-reverse	GGATGAAGCGGAGTCTGGA		
p.c.TERRA-forward	CGGTTTGTTTGGGTTTGGGTTTGGGTTTGGGTTTGGGTT	0.86	[9]
p.c.TERRA-reverse	GGCTTGCCTTACCCTTACCCTTACCCTTACCCTTACCCT		
2p TERRA-forward	TAAGCCGAAGCCTAACTCGTGTC	0.83	[9]
2p TERRA-reverse	GTAAAGGCGAAGCAGCATTCTCC		
18p TERRA-forward	CCTAACCCTCACCCTTCTAAC	0.79	[9]
18p TERRA-reverse	TACCTCGCTTTGGGACAAC		
10q TERRA-forward	GAATCCTGCGCACCGAGAT	1.00	[13]
10q TERRA-reverse	CTGCACTTGAACCCTGCAATAC		
15q TERRA-forward	CAGCGAGATTCTCCCAAGCTAAG	1.00	[13]
15q TERRA-reverse	AACCCTAACCACATGAGCAACG		
XpYp TERRA-forward	GCAAAGAGTGAAAGAACGAAGCTT	1.00	[13]
XpYp TERRA-reverse	CCCTCTGAAAGTGGACCTATCA		
XqYq TERRA-forward	GGAAAGCAAAAGCCCCTCTGAATG	1.00	[13]
XqYq TERRA-reverse	ACCCTCACCCTCACCCTAAGC		
17p TERRA-forward	GAATCCACGGATTGCTTTGTGTACTT	1.00	[25]
17p TERRA-reverse	CCTCAGCCTCTCAACCTGCTTGG		
11q TERRA-forward	CAGACCTTGGAGGCACGGCCTTCG	1.00	[25]
11q TERRA-reverse	CCCTGATTATTCAGGGCTGCAAAG		
**Telomerase activity**			
TS	AATCCGTCGAGGCAGTT	0.97	[23]
ACX	GCGCGG $(CTTACC)_3$ CTAACC		
TSR8	$AG(GGTTAG)_7$		
K1	ATCGCTTCTCGGCCTTTT		
TSNT	AATCCGTCGAGCAGAGTTAAAAGGCCGAGAAGCGAT		[23]
**Telomere length**			
telomere-forward	CGGTTTGTTTGGGTTTGGGTTTGGGTTTGGGTTTGGGTT	0.86	[24]
telomere-reverse	GGCTTGCCTTACCCTTACCCTTACCCTTACCCTTACCCT		
tel-g	ACACTAAGGTTTGGGTTTGGGTTTGGGTTTGGGTTAGTGT		[10]
tel-c	TGTTAGGTATCCCTATCCCTATCCCTATCCCTATCCCTAACA		
36B4u_GC	CGGCGGCGGGCGGCGCGGGCTGGGCGGCAGCAAGTGGGAAGGTGTAATCC		[11]
36B4d_GC	GCCCGGCCCGCCGCGCCCGTCCCGCCGCCCATTCTATCATCAACGGGTACAA		
